# Co-designing obesity prevention interventions together with children: intervention mapping meets youth-led participatory action research

**DOI:** 10.1186/s12966-019-0891-5

**Published:** 2019-12-12

**Authors:** Manou Anselma, Teatske M. Altenburg, Helga Emke, Femke van Nassau, Merlin Jurg, Robert A. C. Ruiter, Janine M. Jurkowski, Mai J. M. Chinapaw

**Affiliations:** 1 Department of Public and Occupational Health, Amsterdam Public Health research institute, Amsterdam UMC, Vrije Universiteit Amsterdam, Van der Boechorststraat 7, NL-1081 BT Amsterdam, The Netherlands; 2 InReturn, Sustainable employability and vitality, Koog aan de Zaan, The Netherlands; 3 Department of Work and Social Psychology, Maastricht University, P.O. Box 616, NL-6200 MD Maastricht, the Netherlands; 4 Department of Health Policy, Management, & Behavior, University at Albany School of Public Health, State University of New York, Albany, New York USA

**Keywords:** Youth-led participatory action research, Intervention mapping, Health behavior

## Abstract

**Background:**

Youth-led Participatory Action Research (YPAR) involves children throughout the process of developing and implementing interventions. Combining YPAR with a structural approach for designing and planning interventions, such as Intervention Mapping (IM), may further improve implementation and effectiveness of interventions. This paper describes how YPAR and IM were combined in the Kids in Action study.

**Methods:**

The Kids in Action study aims to improve health behaviors of 9–12-year old children living in a low socioeconomic neighborhood in Amsterdam, by co-designing interventions with these children. At each of four schools 6–8 children (*N* = 18–24 total per year) and two academic researchers formed participatory groups that met weekly or every fortnight during two school years. An IM expert panel advised the participatory groups on the application of IM.

**Results:**

Following the IM protocol, we conducted a participatory needs assessment with children, parents and professionals, in IM-step 1. In IM-step 2, the IM expert panel constructed matrices of program objectives, and the children provided feedback. In collaboration with children programs were designed and produced using an iterative process during IM-steps 3–4. In IM-step 5, the participatory groups and professional community partners designed the implementation plan. Finally, in IM-step 6, the protocol of the process and effect evaluation – executed by academic researchers with input from children – was developed.

**Conclusions:**

By combining YPAR and IM, several interventions have been developed and implemented, varying from a school water policy to extracurricular sports activities. Sharing responsibility with children was challenging when combining IM with YPAR. In YPAR children are given as much autonomy as possible, while traditional IM development work is primarily done by academic researchers. Strengths in combining IM and YPAR include the involvement of the end-users - children - throughout the process while at the same time developing interventions based on existing evidence. Time-management, a multidisciplinary team, and flexibility are important conditions when combining IM with YPAR. A strong community project group, with professionals who were willing to help children develop and execute their ideas, was an important success factor. This study can serve as an example to other YPAR studies developing interventions using the IM protocol.

## Background

Many children have unhealthy diets and are insufficiently physically active [1–4]. Unhealthy behavior in children is generally more common in low socioeconomic neighborhoods and in certain ethnic groups [5–7]. Interventions promoting a healthy lifestyle in children have shown small effects [8, 9], and those most in need to benefit from these interventions are often hard to include [10, 11]. Few studies involve the perspectives of children themselves in the development of behavioral interventions [12–14]. Involving children in intervention development may improve the relevance, implementation and effectiveness of interventions, as children are experts of their own lives.

In Youth-led Participatory Action Research (YPAR) youth are involved as co-researchers throughout the entire research process [15], from designing the research question to the actual intervention development, implementation and evaluation [12]. This bottom-up approach ensures that the designed intervention meets the needs and interests of the targeted children and increases ownership of the intervention [16]. Children can benefit from the feeling of empowerment and social justice by being involved in YPAR [17–19]. Children learn and develop new skills that are not limited to conducting research, as they for example also develop in public speaking, debating, and critical thinking [15, 20–23]. These empowering skills increase when adults listen to children’s ideas and collaborate with them to turn their ideas into actions [24]. Moreover, instead of merely focusing on problem identification, YPAR focuses on creating solutions and implementing action(s) to improve the situation [15, 17, 20, 25]. Because improvements are more commonly needed in oppressed and deprived communities, YPAR is often used in these communities.

YPAR is an iterative process of planning, acting and observing, in collaboration with children and is influenced by continuous reflection [26]. Therefore, intervention development using YPAR is iterative; problem identification and implementing actions can go hand in hand. For example, when children come up with a good solution they immediately want to implement it. Combining YPAR with a structural stepwise approach for identifying behavioral determinants and evidence-based strategies, such as the Intervention Mapping (IM) protocol, may improve the effectiveness of actions [27, 28]. IM is a systematic approach which ensures that interventions are evidence-based and grounded in theory [29]. IM has previously been combined with community-based participatory research (CBPR) in adults and adolescents [30, 31]. To the best of our knowledge, IM has not been used in combination with YPAR yet.

In the Kids in Action study we use YPAR to involve children from a low socioeconomic neighborhood in developing, implementing and evaluating interventions aimed at the promotion of a healthy lifestyle [32]. To structure the intervention development process and improve the evidence-base and potential effectiveness of interventions, the IM approach was applied to the process of YPAR. This paper aims to describe our approach to structure the intervention development process through combining IM with YPAR within the Kids in Action study.

## Methods

### Kids in action

The Kids in Action study started in 2016 as a collaboration between the Amsterdam University Medical Centers (Amsterdam UMC) and Kids Aktief, an after-school daycare organization located in Amsterdam. The Medical Ethics Committee of the VUmc (2016.366) approved the study. One researcher (MA) was appointed at both organizations. The local government in the northern part of Amsterdam was involved to support embedding the study within existing networks in the neighborhood. Together with these local authorities, a neighborhood in a low socioeconomic area in Amsterdam, the Netherlands, was selected as the study area. In this neighborhood 30% of the 10-year-olds had overweight/obesity in 2016–2018 [33], 31% of the children grew up in a low-income household in 2017 [34], and 38% of the community perceived their neighborhood as unsafe in 2017 [34]. In 2019, the neighborhood had a little over 14,000 inhabitants, with 51% from a non-Western background, mostly from Turkey and Morocco [35]. All four primary schools in this neighborhood were invited and agreed to participate in The Kids in Action study. Control schools were selected from other neighborhoods but with similar characteristics on overweight/obesity, family characteristics and household income [32]. At each intervention school a participatory (YPAR) group was formed consisting of six to eight child-researchers aged 9–12 years (grade 6–8 in the Netherlands) and one or two academic researchers. The participating children and their parents provided written informed consent for the children’s participation. More details on the design of the study can be found elsewhere [32]. The collaborations within this study include:

- **YPAR groups**: Six to eight child-researchers and one or two academic researchers from the Amsterdam UMC;
- **Planning group**: Academic researchers from the Amsterdam UMC, youth policy managers of the local government of the North of Amsterdam, child-researchers from YPAR groups;
- **IM expert panel**: Six academic researchers with expertise in applying IM; and
- **Community project group**: Academic researchers from Amsterdam UMC, youth policy managers of the local government in the North of Amsterdam, representatives of other organizations working with children in the neighborhood such as social workers, teachers and principals of primary schools.

#### Participatory meetings with children

During three school years (2016–2019), the YPAR groups met weekly or every fortnight to work on the study, depending on the preferences of the school. The meetings were facilitated by one or two academic researchers. When there were two facilitators, one researcher led most of the meetings and the other made observations, took notes, monitored the planning of the meeting, asked questions if she felt clarification was needed and helped the child-researchers with their assignments. The meetings were organized at the children’s school in a classroom or teacher’s room. The academic researchers established a collaborative atmosphere in the meetings in which relationships and trust were built with the children, for example by reorganizing chairs and tables, setting ground rules in the first meeting, and starting the meetings with an icebreaker/game and making jokes. If needed, children could work in smaller groups somewhere else in the room or school to feel less of the group pressure or increase their focus.

#### Intervention mapping (IM)

IM provides program planners with a framework to structurally develop theory-based interventions [29]. IM structures this process by providing six steps that support program planners towards effective decision making, considering the community as well as scientific theories: 1) Creating a logic model of the problem, 2) Describing the program outcomes and objectives, and creating a logic model of change, 3) Designing the program, 4) Producing program components and materials, 5) Developing the program implementation plan, and 6) Planning for evaluation. A detailed description of the IM steps are described elsewhere [28, 29, 36] and in Additional file 1 an overview of the IM steps and their application in Kids in Action can be found. An IM expert panel was installed to 1) discuss how the IM process could be conducted in close collaboration with the child-researchers; and 2) conduct the parts of IM that would be inappropriate for the child-researchers. The panel met twice, and provided feedback on the first three steps. In the first meeting (March 2017) the outline of the study was discussed and in particular the design of the first step of IM. Additionally, the panel decided which parts of IM would be conducted by the panel and which parts by the YPAR groups, based on the complexity of the IM parts and how and if this could be simplified in the YPAR groups. During the second meeting (April 2017) the outline of the matrices of step two were discussed and how to proceed with these matrices in the YPAR groups.

Below we explain step-by-step how the combination of IM and YPAR was applied. Each IM step begins with the key objectives of the tasks in that step, followed by the practical application in the Kids in Action study.

### Step 1: logic model of the problem

The first step of IM includes establishing a planning group, conducting a needs assessment, assessing the community’s context, linking this to program goals and creating a logic model of the problem [29].

#### Partnerships for YPAR

The academic researchers approached the local government in the north of Amsterdam and discussed which low socioeconomic neighborhood could benefit from participating in this study. The local government then selected a neighborhood and helped to create partnerships in the community. We created sustainable partnerships among the community, experts in the field of IM and YPAR, and the children. The selected neighborhood already had a community project group, representing different community organizations such as youth social work, a theater group, and an after-school daycare. This community project group discussed and assisted each other in projects and new project ideas. By joining this existing community project group, their network could be used and therefore it was not needed to form a new project group. The community project group supported the study and was willing to collaborate in the process of intervention development.

Schools’ preference guided the selection of children and planning for the YPAR groups. For example some schools preferred that teachers selected children for the YPAR groups while at other schools children could sign themselves up. Depending on the schools’ preferences, meetings were held for one hour during school hours, or for 45 min after school hours followed by a 45-min sports activity, to motivate children to come to the meetings in their free time. Each school year YPAR groups changed as children of the highest grade left the school and new children could fill these spots. Therefore after the first year, the YPAR groups included children participating for the first time as well as children who already participated in the previous year.

#### Participatory needs assessment

The needs assessment was conducted over two school years in collaboration with the YPAR groups and the community project group. At the start of the Kids in Action study in 2015, the academic researchers collaborated with children in YPAR groups, joined informal meetings with parents, and interviewed professionals (policy makers working on sports and/or child development and health behavior, social workers working with youth and a teacher) and parent-child dyads, to gather data for the needs assessment [37].

In 2016, the following school year, newly formed YPAR groups conducted their own research to verify findings from the previous year. To prepare the child-researchers for conducting their own research, one of the first YPAR meetings was dedicated to basic research principles and methods. The child-researchers for example learned about privacy, asking permission, acting professional, conducting interviews, focus groups, and taking pictures and other potential research methods and techniques. Next, the YPAR groups formulated research questions regarding why children do not regularly participate in sports and other physical activities and why they often choose unhealthy dietary options. The YPAR groups were divided into smaller subgroups and each of these groups chose one or more research questions they wanted to answer. An example of a research question was: “In what way is the neighborhood encouraging or discouraging children to play outside?”. The subgroups decided together with the academic researchers upon an appropriate research method to answer the research question(s). The academic researchers facilitated the necessities for the research. Chosen methods were: interviews, questionnaires (on paper and online), photos, and focus groups. In the following meetings the subgroups organized their data on large sheets of paper assisted by the academic researchers and presented their conclusions to the other subgroups.

To achieve data saturation, the IM expert panel advised to perform focus groups with the child-researchers enriched with some of their peers. These focus groups were co-organized by child-researchers from the YPAR groups. In these focus groups an ordinary school day was discussed from the moment the children wake up until they go to bed, with the focus on which physical activities they performed and their dietary intake. Additionally, potential underlying determinants were discussed. At three schools two focus groups were conducted (five to eight children per group); one on dietary behavior and one on physical activity. At one school both topics were combined in one focus group, because only nine children were present. The children placed notes of all their activities and their intake of foods and drinks on a timeline. The academic researchers asked clarifying questions and initiated discussions by stimulating the children to form clusters of related behaviors. Clusters of dietary intake were breakfast, snacks during the school break, lunch, after school snack, dinner and evening snack. Clusters of physical activities were activities before going to school, transport to school, playing outside during the school break, playing outside after school and going to a sports club. The academic researchers analyzed the focus groups and in the next meeting the findings were discussed within the YPAR groups to explore the credibility of the researcher’s analyses [38].

### Step 2: program outcomes and objectives and logic model of change

The second IM step specifies what needs to be changed and by whom in order to reduce the health problem [29]. Expected program health outcomes are stated, and behavioral and environmental outcomes are divided into performance objectives. Performance objectives and changeable determinants of the behavior in matrices are crossed, thereby creating change objectives. Matrices of these change objectives are created for each relevant ecological level for the intervention.

In the current study, the academic researchers constructed the matrices based on the data collected in the needs assessment with multiple feedback rounds from the IM expert panel. Matrices were created for multiple stakeholders: children, parents, local government, and school staff.

Together with the IM expert panel we decided not to include the child-researchers in the creation of the matrices as this would require educating them on behavioral theories, which we perceived as unrealistic and inefficient [24, 25]. The IM expert panel designed performance objectives that represented the goals that needed to be reached to accomplish the overall program goals. The YPAR groups were asked to give feedback on the matrices by showing them a list of all performance objectives of the children’s matrix, described to them as goals. The IM expert panel decided not to discuss the change objectives with the children as there were too many.

### Step 3: program design

In the third IM step program ideas are generated with the planning group: theoretical methods that can be linked to the program goals are determined and form the program methods [29, 39]. Subsequently, methods are translated into practical applications that address the change objectives and fulfill the theoretical parameters for effectiveness.

After agreeing on the final performance objectives (in step 2), each member of the YPAR groups could vote for three dietary performance objectives/goals he or she wanted to work on and three physical activity performance objectives/goals. The YPAR groups decided to work on the most popular goals in subgroups, each subgroup working on one goal. The child-researchers discussed in their group how they could achieve the goal. They did not directly have to take feasibility into account, but could pretend they were somebody else, for example: “If I was the mayor of Amsterdam, how would I reach this goal?”. The child-researchers did not think of practical intervention strategies straight away, but first mentioned change objectives; they knew what they wanted to change but did not directly know how. The academic researcher asked questions to help the child-researchers think of practical intervention ideas, based on the data from the needs assessment. For example, “In the focus groups we discovered this [*the feeling that the neighborhood is not safe*] was a problem, how can we take that into account?”. Furthermore, the academic researcher had the change objectives ready to help children incorporate those in their intervention ideas. The YPAR groups formulated a list of 20–30 intervention ideas for the performance objectives they were working on and voted for the best ideas. The researcher explained that ideas that were not chosen at that moment, could be worked on at a later stage. To identify feasible and effective methods for intervention strategies the academic researchers checked the IM literature describing effective strategies [29]. For this step the IM expert panel provided advice on how to match the program ideas to theoretical/effective methods of change and how to structure the program ideas, strategies and implementation plans. Additionally, the researchers conducted a literature review summarizing evidence-based intervention strategies in similar target groups (protocol registered in PROSPERO: registration number CRD42016052599). These two steps ensured that evidence-based components were included in the developed actions. If there were no evidence-based strategies that could be linked to children’s intervention ideas, the YPAR groups discussed if the idea could be shaped differently to include evidence-based strategies. From the list of potential effective ideas, the YPAR groups chose one or two ideas they wanted to develop into a detailed program plan. Developing a detailed program plan took three to four meetings.

### Step 4: program production

In the fourth IM step the program structure and organization from step 3 are specified [29]. Production plans for the intervention materials are developed and piloted.

In this step, program materials and components were developed together with the planning group and professionals from the community project group. This step was intertwined with the previous step, as the child-researchers already came up with input for materials and components while thinking of program ideas. For example, in step 3 the YPAR groups had the idea of organizing extra sports activities and discussed which objectives they would address with these activities. At the same time, they discussed which sports they wanted to organize, where and when. To help the child-researchers develop a detailed program plan for each of their programs, the academic researchers asked questions to help them think of all aspects of a program plan. For example “Who is going to guide that [*the sports activity*]?”, “What materials do you think you need?”, “How are we going to make sure many children sign up?”, or “Do you think you can execute this by yourself, or do you need to ask others for help?”. During the YPAR meetings the groups worked on developing materials, ideas for promotion materials, and asked their peers what would convince children to participate. Next, the YPAR groups chose one or two program plans that they pre-tested before the summer break in 2017. At one school the pilot was canceled because of bad weather conditions. The YPAR groups evaluated the pilots and if needed adapted the program plans for the actual implementation.

### Step 5: program implementation plan

In the fifth IM step a program implementation plan is made [29]. Potential adopters and implementers are identified, matrices of change objectives are developed for adoption, implementation and maintenance of the program, and requirements for successful implementation are noted.

The YPAR groups developed the implementation plan together with relevant stakeholders, such as the school principal, sports coaches, and social workers. Meetings with stakeholders were mostly held without the child-researchers, as planning these meetings together with children was rarely possible because of their schedule and logistics. When child-researchers needed help from a stakeholder, they discussed with the academic researchers who they thought was suitable to contact. Preferably, this was a partner from the community project group, because they already knew the study, the neighborhood and the target group. The main researcher presented the implementation plan made by the YPAR group to the community partner to discuss intervention adoption, implementation requirements and sustainability of the intervention. The academic researcher shared the community partner's view with the YPAR groups and if they agreed the new ideas were incorporated in the implementation plan. The implementation plan was finalized in agreement with the YPAR groups and the community partners.

Implementation plans were developed for and implemented one intervention at a time. Once successfully implemented, the YPAR groups could decide on a new intervention to work on (steps 3–5). In this way intervention development remained a continuous process where multiple performance objectives could gradually be realized. If different YPAR groups had similar intervention ideas, organization of the intervention was shared. For example if the intervention was available for children within the whole neighborhood, one YPAR group was in charge of recruitment while the other group was responsible for the content. If a similar intervention was organized at two schools on two separate days, one implementation plan was created where the YPAR groups could incorporate their own creativity in the execution.

### Step 6: development of evaluation plan

In the last IM step, the process and effect evaluation is developed, for which indicators, measures and designs have to be developed [29].

The Kids in Action study includes a process and effect evaluation. For both the process and effect evaluation of the Kids in Action study, we asked child-researchers to provide input for designing the evaluation. The evaluation itself was conducted by academic researchers.

## Results

This section describes the results per IM-step. At the end of each IM-step one example is described in more detail.

### Step 1: logic model of the problem

**Fig. 1:**
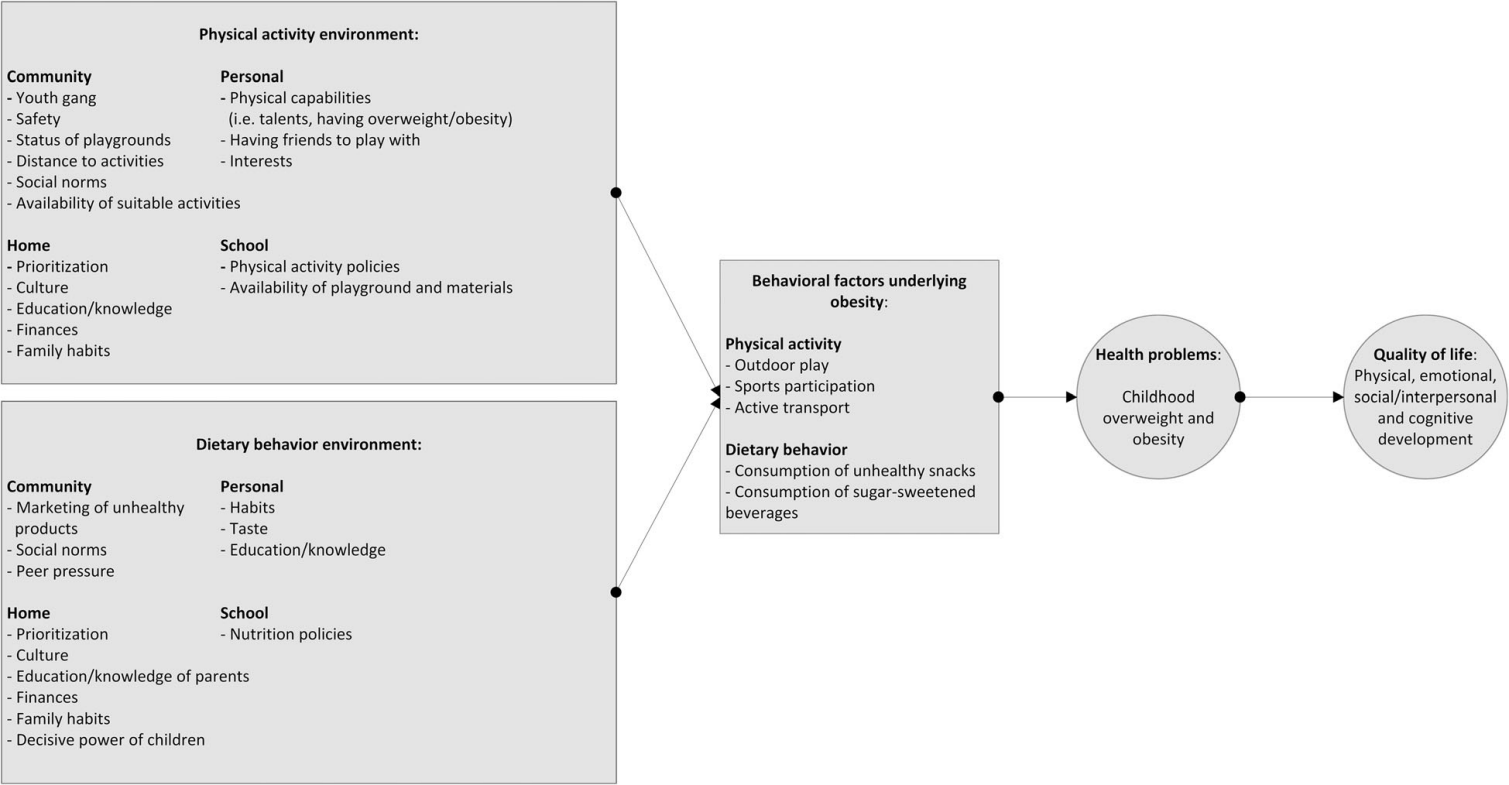
Logic model of the problem

A detailed description of the results of the needs assessment can be found elsewhere [37]. The two main identified causes for unhealthy behavior were insufficient physical activity and unhealthy dietary behavior. Underlying hypothesized determinants for insufficient physical activity included perceiving the neighborhood as unsafe for example due to a youth gang in the community. This is also related to parents not wanting their children to go to activities by themselves, and the perception that all organized activities that were not at school or close to the home are too far. Furthermore, a lack of finances and knowledge, social norms and culture were important underlying determinants for children being insufficiently physically active and also for unhealthy dietary behavior. For the latter, difficulties with changing habits were also mentioned and children having too much decisive power. If they for example do not like a meal with vegetables, parents will cook something else. The results of the needs assessment were used as input for the Logic Model of the Problem (see Fig. 1). This model depicts which aspects of the physical activity and dietary behavior environment impact the behavioral factors underlying overweight/obesity in children. Based on the needs assessment, four program goals were set: 1) Children participate in more outside play, 2) Children participate in more organized sports activities, 3) Children drink fewer sugar-sweetened beverages, 4) Children eat fewer unhealthy snacks. Needs that were identified related to physical activity were for example more supervised sports activities at a nearby location and for a low price. Related to dietary behavior the most important expressed need was more education on healthy dietary behavior.

*An example of a health problem identified by the YPAR groups in the needs assessment was that children drink a lot of sugar-sweetened beverages* [37]*. Many schools in Amsterdam have a policy that children can only drink water at school. Not all schools adhere strictly to this policy, and one of the participating schools had not implemented this policy. At this particular school, children identified that a lot of children drank sugar-sweetened beverages at school.*

### Step 2: program outcomes and objectives and logic model of change

**Fig. 2:**
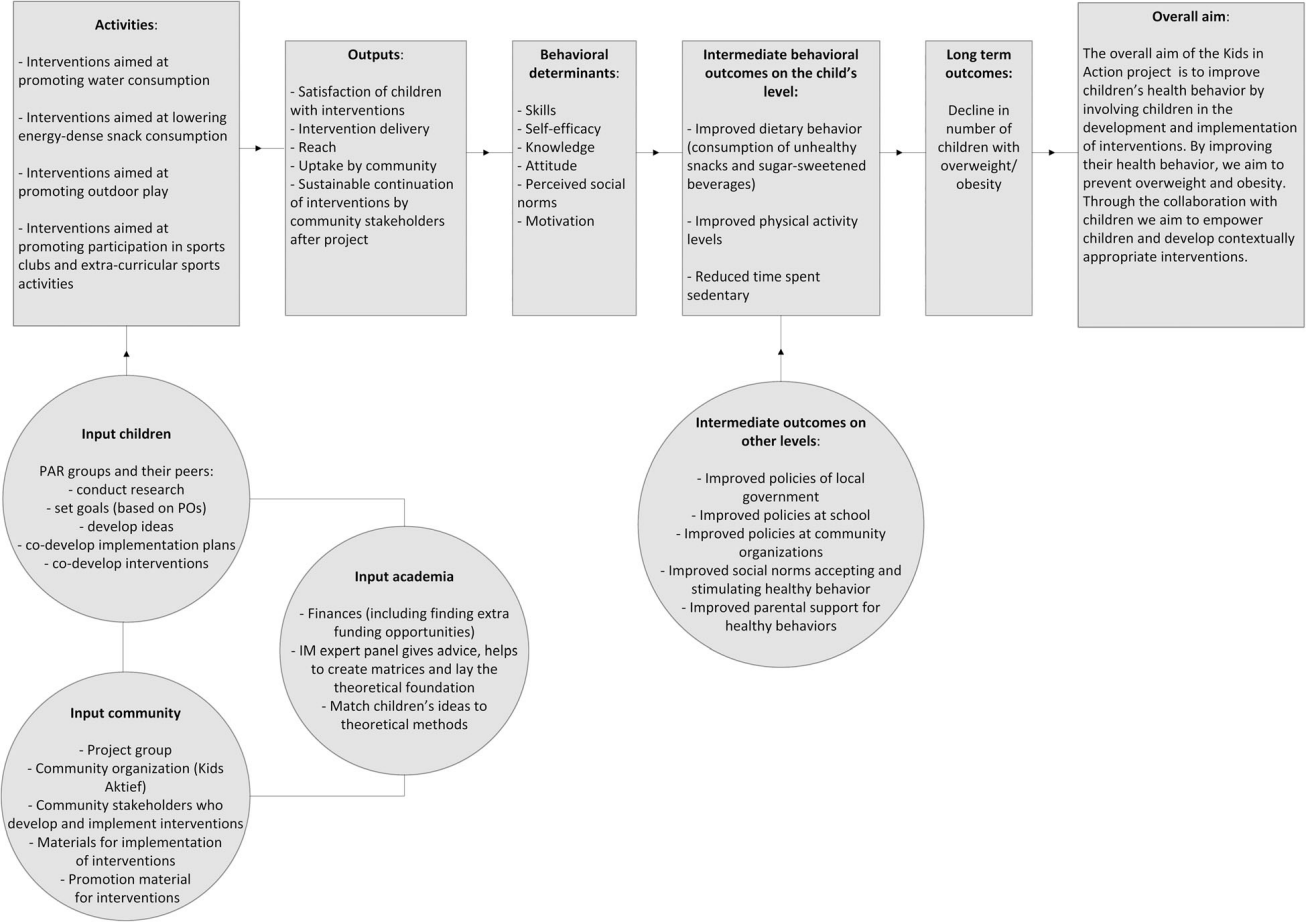
Logic model of change

Based on the needs assessment, the academic researchers created a logic model of change (see Fig. 2). The PAR groups and their peers provided intervention ideas related to the four program goals. The community partners were identified as important for implementation and creating support for adoption. Lastly the input of academic researchers was needed for a theoretical foundation of IM and YPAR and applying for extra funding for the developed interventions. The logic model of change further shows that via the output (e.g. satisfaction of children with intervention and uptake by the community), behavioral determinants will improve (e.g. skills, knowledge, attitude), leading to intermediate behavior change outcomes (improved dietary behavior, physical activity levels and reduced sedentary time) resulting in the decline in childhood overweight/obesity.

Next matrices of change objectives were developed. Tables 1 and 2 present examples of performance and change objectives on the level of children, parents, local government and school staff, related to two of the many intervention ideas of the YPAR groups: the Olympic sports event (Table 1) and cooking classes (Table 2). Determinants that were crossed with the performance objectives were skills and self-efficacy, knowledge, perceived social norms and attitude, leading to the change objectives. The YPAR group provided feedback on the performance objectives of the children’s matrices, described as goals. As expected the child-researchers brought up performance objectives for other stakeholders, especially their parents. For example the performance objective ‘Children only drink water at school’ was identified as highly dependent on how strict teachers are with maintaining the rules. Overall, children agreed with the performance objectives. Mainly the performance objectives focusing on healthy dietary behavior were judged important but not appreciated, as children for example enjoyed soft drinks and were not willing to lower this consumption.

**Table 1:** Examples of performance and change objectives related to the Olympic sports event

Program goal: More children participate in sports activities				
Performance objective children	Skills + self-efficacy	Knowledge	Perceived social norms	Attitude
Children participate in sports activities of their own preference: - Children ask their parents for financial aid to participate in a sports activity - Children choose the sports activity that they like most - Children structurally participate in a sports activity of their own preference	- Children participate in a sports activity independent of where their friends are going - Children demonstrate confidence in participating in sports activities independent of their friends	- Children know the sports activities that are on offer - Children know the sports activities that they prefer - Children know alternative financial possibilities to cover their sports participation	- Children recognize that peers participate in sports activities of their own preference	- Children have positive feelings towards participating in sports activities
Parents facilitate participation of their children in organized sports activities	- Parents demonstrate that they can find alternative financial possibilities to cover sports memberships	- Parents know the advantages of sports participation - Parents know the alternative financial possibilities to cover their child’s sports participation	- Parents acknowledge that it is nothing to be ashamed of to ask for financial aid for sports participation coverage	- Parents express positive feelings towards their child’s sports participation
Teachers and school management stimulate children to participate in sports activities	- Teachers have confidence they can stimulate children to participate in sports activities. - School management demonstrates that they can offer sports activities that are of interest to children	- Teachers know which sports activities for children are organized in the community - Teachers know which alternative financial possibilities there are for children to cover sports memberships	- Teachers and school management recognize that children at other schools are being stimulated to participate in sports activities	- Teachers and school management feel positive about children participating in sports activities
Policy makers increase the capacity of popular sports activities organized by the local government		- Policy makers know the sports activities for which higher capacity is wanted		- Policy makers feel positive about making more sports coaches/ materials/centers available for children’s sports activities

For each level – children, parents, schools, local government – one performance objective and its change objectives are given

**Table 2:** Examples of performance and change objectives related to the cooking classes

Performance objective children	Skills + self-efficacy	Knowledge	Perceived social norms	Attitude
Children always eat a healthy (amount of) breakfast in the morning before they go to school	- Children demonstrate that they can make their own breakfast	- Children know different kinds of healthy breakfast (for example for children for whom bread is too heavy, they describe alternatives) - Children know the importance of a healthy breakfast	- Children recognize that peers have a healthy breakfast	- Children have positive feelings towards having a healthy breakfast every morning
Parents automatically offer healthy snacks to their children	- Parents demonstrate the confidence that giving healthy snacks is good for their children’s health	- Parents know that giving healthy snacks are good for their children’s health	- Parents recognize that other parents give their children healthy snacks	- Parents recognize that healthy snacks are also enjoyable/a good alternative/good for their children’s health and are tasty
The school management checks the adherence of the policies supporting fruits and vegetables during break time and healthy birthday snacks with teachers and in classrooms	- The school management demonstrates how they can involve teachers in adhering to the policies		- The school management recognizes how other schools successfully implement and adhere to the health snacking policies	- The school management acknowledges the importance of the policies and what they stand for - The school management feels positive towards strict adherence of the policies
The municipal health services regularly checks all schools on the progress and implementation of policies supporting fruits and vegetables during break time and healthy birthday snacks	- The local government demonstrates that they can convince schools to incorporate and adhere to policies supporting fruits and vegetables during break time and healthy birthday snacks	- The local government knows how they can work together with schools towards successful implementation of policies supporting fruits and vegetables during break time and healthy birthday snacks		

For each level – children, parents, schools, local government – one performance objective and its change objectives are given

In the community project group, an academic researcher presented the performance objectives for the local government and community partners who then provided feedback. The performance objective ‘Children only drink water at school’ was extensively discussed. The community project group supported this performance objective and committed to only serving water to children and educate them on reasons why, thereby also trying to change the social norms in the community.

### Step 3: program design

The YPAR groups received a list of performance objectives from the children’s matrices – phrased as goals – and could choose which goal they wanted to work on. Generally, children were more interested in physical activity than dietary behavior. They were encouraged to choose performance objectives for both topics and also work out ideas for both topics. Table 3 presents the selected performance objectives and initial intervention ideas. Below an example is provided of a program idea which relates to the example in the previous steps:

*Two boys from the YPAR group at the school without a water policy chose to work on the performance objective ‘Children only drink water at school’. A result from their own research during the needs assessment was that a lot of children drink sugar-sweetened beverages at school. They also knew that other schools had a water drinking policy, prohibiting sugar-sweetened beverages at school. Therefore, their idea was to introduce a water policy at their school.*

**Table 3:** Overview of goals, initial ideas, change objectives and the developed intervention activities

Goals retrieved from performance objectives	Initial ideas that were voted for	Examples of related change objectives on the children’s level	Intervention activities and implementation status
Children always eat a healthy (amount of) breakfast in the morning before they go to school	Create a lesson series on what a healthy breakfast is and combine it with quiz elements, then children will like it more and remember the message (school 1, 2, 3)	Children know different kinds of healthy breakfast Children recognize that peers have a healthy breakfast Children have positive feelings towards a healthy breakfast	Cooking workshops (implementation plan in Additional file 3): at first of a duration of 1 month, then they were taken over by a community partner who organizes it throughout the year Quiz at school (once) and regularly recurring at after-school activities
Children eat less unhealthy snacks at school	Organize a competition at school where you can win a prize if you take healthy snacks and lunch to school (every month a different prize) (school 2)	Children describe what healthy snacks are Children describe the importance of eating enough fruits and vegetables every day Children recognize that peers eat fruits and vegetables during breaks	Healthy snacks and lunch competition at school during 3 months Cooking workshops: at first of a duration of 1 month, then they were taken over by a community partner who organizes it throughout the year
Children drink only water at school	Create a water fountain at the school playground where you can always drink water during and after school (school 1, 3) Start a policy at school that children can only drink water (school 3)	Children recognize that peers only drink water at school It is a habit that everybody has a water bottle with them Children demonstrate how they can make water taste better (e.g. by adding fruits)	Water fountain installed at one school, together with a policy that children can only drink water at school (school 3)
Children drink tea without sugar	Create a lesson series where children learn to drink tea without sugar, then they will get used to it and like it (school 1)	Children acknowledge that tea with sugar is not healthy Children demonstrate how they can make tea taste better, without adding sugar Children demonstrate the confidence that they can break tea drinking habits	Cooking workshops: at first of a duration of 1 month, then they were taken over by a community partner who organizes it throughout the year
More children play outside (actively)	Make playgrounds with equipment suitable for children of different ages and teach children active games that they can play there (school 1, 3)	Children describe active games that they can play Children demonstrate different kinds of active games that they can play at a playground Children recognize that peers play fun active games outside	The local government adjusted several already existing playgrounds After school activities were organized for 4 months where children learned new active games that they could play In after-school activities of this project and also of community partners, more focus was placed on active games that children themselves could play without a lot of extra materials
More girls participate in after-school sports activities	Organize more girls-only activities, and ask girls what kind of activities they like (school 1, 2)	Girls recognize that physical activity is important Girls see other girls having fun in sports activities Girls have positive feelings towards the activities that are organized for them	A weekly girls-only activity was started for the duration of 2 years
Children participate in sports activities of their own preference	Let children themselves co-organize activities and make sure there are good coaches to supervise, so they will like it more (school 1, 2, 3)	Children know the sports activity that they prefer Children demonstrate confidence in participating in sports activities independent of their friends Children like participating in sports activities (that they have organized)	The Olympic sports event consists of yearly after-school sports activities followed by a sports tournament for the four schools in the community (implementation plan in Additional file 2) Children co-organized all intervention activities In after-school activities of this project and also of community partners, the aim was to give children a positive sports experience In after-school activities of this project and also of community partners, children could co-decide on the activities that were offered
Less children are behind a screen after school (computer, television, phone)	Organize more after-school sports activities and events so children are stimulated to play outside (school 1, 2)	Children describe the advantages of playing outside over screen time Children acknowledge that screen activities are for the late afternoon/evening Children perceive that peers are not behind a screen after school	After-school sports activities were organized during 4 months where children learned new active games that they could play In after-school sport activities of this project and also of community partners, more focus was placed on activities that children themselves could play without a lot of extra materials The Olympic sports event consists of yearly after-school sports activities followed by a sports tournament for the four schools in the community (implementation plan in Additional file 2)

### Step 4: program production

The YPAR groups developed detailed program plans for one or two of their program ideas. For some YPAR groups it was difficult to go into detail or find solutions for barriers. One YPAR group for example wanted to organize tasting sessions to stimulate their peers to taste tea without sugar. But as children did not have access to the school’s kitchen, lack of access to hot water was a barrier. As can be seen in Table 3, eventually multiple performance objectives led to similar practical applications. This happened iteratively as in one YPAR group, two or three subgroups worked on different ideas. At the end of each session they had to explain to the other subgroups what they had worked on to inspire each other and form new collaborations. Also, if different YPAR groups worked on similar ideas, the researchers shared this information. This led to all YPAR groups working on the cooking workshops and the Olympic sports event. For most interventions, meeting-by-meeting more detail was added to the production plan. Below an example is provided of how a production plan was shaped and how a program was piloted:

*Two boys decided together with the principal to start a 3-week pilot before the summer holiday so children and parents would get used to drinking only water at school. To motivate children, the class with the most children drinking water during the pilot could win an extra 15 minutes of outside play. The two boys made posters where teachers could keep track of how many children only drank water. The boys were also responsible for weekly collecting the posters and counting the scores. Lastly, the principal wanted to inform the parents about the new policy, and invited the boys to write a paragraph for the newsletter, explaining their motivation for introducing the policy.*

Not all program ideas were pilot tested, for example due to lack of finances for the execution. For example, finances were obtained to organize the Olympic sports event, but this did not include pilot testing. Since the YPAR groups organized multiple sports activities, they could learn from the iterative evaluations of these activities and thereby pilot test certain elements of the Olympic sports event. The YPAR groups could for example monitor which sports were most popular and whether children appreciated yellow and red penalty cards for lack of sportsmanship (see Additional file 2 for the implementation plan of the Olympic sports event).

### Step 5: program implementation plan

In this step the implementation plans were developed. Additional file 2 and Additional file 3 describe the implementation plans of the Olympic sports event and the cooking workshops. The YPAR groups made their own overviews and decided on the division of tasks. The researchers made a separate overview, which is depicted in Additional file 2 and Additional file 3, to make sure all the practical applications were covered and the interventions and its strategies were linked to methods and determinants. Below an example is provided on how an implementation plan was developed:

*The YPAR groups wrote down everything they had to do and who they needed to successfully implement the water-policy (for example teachers, the principal, parents, experts). The most important thing was approval of the principal. Together with the main researcher the children discussed their plan with the principal, who was enthusiastic and willing to help. The researchers convened with policy makers who had experience with implementing water-policies at schools as part of the Jump-in intervention* [40]*. The policy makers liked that teachers had to keep track of children’s water consumption and that classes had to compete against each other. The policy makers emphasized the importance of including parents throughout the process, for example by organizing workshops for parents. The policy makers shared posters and leaflets with information on the amount of sugar in sugared-beverages and advice on how to make water taste better (e.g. add fruit). Lastly they advised to have a celebratory opening and have multiple activities with water to give it a positive image.*

### Step 6: development of evaluation plan

**Fig. 3:**
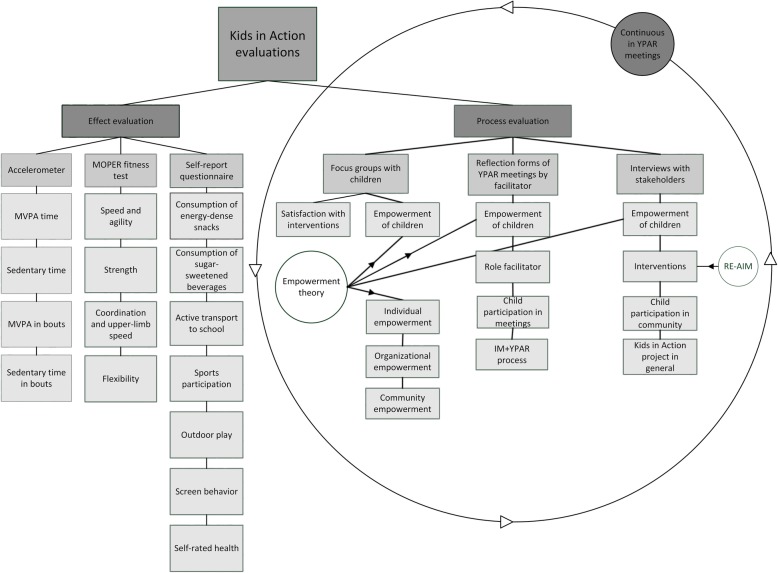
Overview of all evaluations within the Kids in Action studyWith legends: Abbreviations: MVPA: Moderate-to-Vigorous Physical Activity; RE-AIM: reach, effectiveness, adoption, implementation, maintenance

In contrast to the previous steps where children co-created the first drafts, the academic researchers developed a first draft for both the effect and process evaluation. Next, children were asked to reflect on the proposed measures and to suggest potential additional evaluation outcomes or methods. Figure 3 presents an overview of the effect and process evaluation of the Kids in Action study. The results of the process and effect evaluation will be published separately.

The primary effect outcomes were based on the target behaviors identified in the needs assessment and related outcomes: dietary behavior, physical activity, sedentary behavior, physical fitness, and self-rated health. Dietary behavior, sports participation, screen time and self-rated health were measured using a questionnaire with validated items from the ENERGY-child questionnaire [41], the DOiT questionnaire [42], and the EuroQol [43]; physical activity and sedentary time were measured using accelerometers; and neuromotor fitness was measured using the MOPER fitness test [44]. The effect outcomes were measured at baseline (throughout first school year; 2016–2017) and at the end of the two subsequent school years (April–May 2018 and March–May 2019) at four intervention schools and four control schools. More details on the evaluation design are described in the protocol paper [32]. All YPAR groups agreed with the proposed outcome measures selected by the academic researchers, and did not want to include additional ones.

We applied a qualitative process evaluation because we were interested in how the respondents perceived the IM and YPAR process and the developed interventions. The process evaluation was designed by the academic researchers to suit the YPAR process and included three main focus points: the IM + YPAR process, children’s empowerment, and the experiences of children and professionals with Kids in Action and its interventions. Throughout this study, data on experiences and empowerment of children was gathered through reflection forms which were filled in by the academic researcher at the end of every YPAR meeting. The reflection form included a summary of the meeting and a reflection on the collaboration with children and the group process. These reflection forms will be used for a detailed evaluation of developing and implementing interventions together with children using IM and YPAR. Yearly focus groups were organized to evaluate effects on individual, organizational and community empowerment as we wanted to know how each of these components developed during the study [45, 46]. The focus groups were held with children from the YPAR groups as well as with other children from the participating schools. The focus groups included two exercises: one exercise evaluated individual empowerment and another exercise evaluated organizational and community empowerment [32]. As part of the second exercise children’s judgement of the developed interventions was also discussed. Furthermore, children evaluated the reach of, and experiences with, the interventions they implemented using their own evaluation methods, as shown in the following example:

*During and after the pilot the principal was asked about his/her experiences regarding the implementation of the water-policy. In the following school year, the new YPAR group designed a short survey, in which questions were asked regarding children’s experiences with the water policy.*

At the end of the study, as part of the process evaluation, professionals from the community project group were interviewed about their experiences with the Kids in Action study and the developed interventions. We based the interview guide on the RE-AIM framework. The RE-AIM framework is designed to structurally evaluate top-down interventions on five dimensions: reach, effectiveness, adoption, implementation, and maintenance [47]. However, as the framework is not created to evaluate the whole process of YPAR, we used RE-AIM as a basis for the process evaluation and added topics related to the process of YPAR. Respondents were for example questioned regarding the reach of the interventions, the perceived effects of the interventions, embedding within and implementation by the community project group and thoughts regarding the continuation of the interventions after the Kids in Action study. The interview guide was completed by adding questions regarding the evaluation of the participatory process with children and the Kids in Action study as a whole; for example questions were asked about the perceived effects on children’s participation in the study and their experiences with the collaboration and communication during the study.

## Discussion

This paper describes how IM and YPAR are combined in the development of health promoting interventions together with children. By combining a structural approach for designing and planning interventions and evidence-based theories with participation of the target group, we aimed to develop interventions that are relevant and attractive to the target group and thereby more implementable and effective than top-down implemented interventions.

We experienced that combining YPAR and IM has multiple strengths. In YPAR studies it can be difficult to include existing evidence in developing interventions, as children want to act really fast. As a researcher it can be difficult to not give in to the children’s enthusiasm. The IM approach provides the needed structure to take a step back and first reflect on ideas and relate them to theory [48]. Within this process, children learned to structure their ideas, set up research questions, contact and communicate with stakeholders, reflect on feasibility of ideas and voicing their opinion. This was also appreciated by decision makers (policy makers, school principal), who admired the children’s ideas and drive for change. One of the core elements of Participatory Action Research is that it “seeks to understand and improve the world by changing it” [17], where understanding of the local context happens by valuing the expertise of the target group. Through this understanding, interventions can be developed that might better fit their local context. Our process evaluation will give us insight in whether this was true.

In our study we experienced that the participation of the target group and community partners from the start of the study was very valuable, and that children from 9 to 12 years old were capable of actively participating in a study that combined YPAR and IM. Similar to collaborating with adolescents and adults [30, 31], the present study demonstrated that children aged 9–12 could also successfully execute certain IM-tasks, such as looking at changeability and feasibility of program ideas, prioritizing them, thinking about who to involve and possible barriers. The perspectives of children on both the problem as well as solutions provides a thorough understanding of the problem and stronger ownership of interventions as they have co-developed them. In this way more attractive, relevant and thereby effective interventions may be developed. Future research should evaluate if interventions that are developed by combining IM and YPAR are more sustainable and effective in improving health behavior in the long term than only using one of both methods.

The iterative manner in which we combined IM and YPAR provided us with challenges and lessons learned. The main challenge we experienced in combining IM and YPAR was the balance in sharing responsibility with children. In YPAR it is preferred to have as much autonomy for children as possible [49]. However, by aiming to apply a structural stepwise approach for identifying behavioral determinants and evidence-based strategies, some parts of the Kids in Action study were conducted without participation of the child-researchers such as the development of the matrices. Future studies may experiment with even more child responsibility, for example, exploring alternative fun and energetic ways to involve children in the development of the matrices, or designing child-appropriate IM workshops [30]. Studies combining participatory research and IM with adults have shown opportunities towards more power and shared-decision making concerning the execution of the IM steps and knowledge of the theory [30, 31]. Another opportunity could be to include parents or other community stakeholders for these more theoretical steps. Involving these stakeholders earlier in the process could also have a positive effect on implementation and maintenance of the interventions.

The second challenge was collaborating with children and all community partners. On the side of the academic researchers, IM expert panel and schools, it required time-management and flexibility to conduct a study in which YPAR and IM was applied. Researchers have multiple roles and responsibilities that may limit their ability to spend extensive time on YPAR meetings. The IM expert group needed to be flexible as preferably feedback was provided to the YPAR groups within a week. It was helpful to organize meetings during school hours, as children tend to be more focused. However, this does ask a lot of schools because the work is outside of the required curriculum. Investing in a transparent and mutually beneficial relationship with schools is therefore essential for successful implementation of YPAR [50–52]. But the most important relationship is that with the children, as the entire research process is dependent on the availability and motivation of children [24]. It is important to recognize the potential limitations of children’s ability, which depends on their cognitive developmental stage. It can be challenging to have children work efficiently on highly complex intellectual tasks for the length of time needed to work through IM-steps. This makes it difficult to anticipate how long certain IM steps will take. When conducting such a process with adults or adolescents the process is still iterative and flexible, but it is a bit easier to anticipate the execution of the process [30, 31]. In our study we experienced that children were very enthusiastic and could rush through theory, or found certain steps difficult which then resulted in needing multiple meetings to work through details. As this also differed between groups and exercises, it was difficult to decide when to give children responsibilities to work on something by themselves, as we did not have unlimited time.

An important success factor in the Kids in Action study was that the study collaborated with a multidisciplinary planning group consisting of a community-based organization familiar with the neighborhood. This facilitated the contacts with the schools and other stakeholders. By being part of the community project group, we could add items of our interest to the agenda in meetings which resulted in more involvement and support of partner organizations. Establishing this position was time-consuming at the start of the study, but it strengthened the study and network in the area and facilitated implementation and sustainability of actions. Through this network the researcher could also easily pitch the children’s ideas to partners who in turn could help the children with development and implementation [16].

## Conclusions

We experienced that combining YPAR and IM strengthened the development process of interventions. The YPAR methodology supports that interventions match the needs of the target group; while IM promotes that intervention design is structured and based on existing evidence. Success factors for combining IM and YPAR are a multidisciplinary team with experts in both approaches, willingness from academia and community partners to collaborate with children from the community, and flexibility from all stakeholders. A strength was that facilitators in the YPAR groups were flexible, resulting indynamic sessions where children could be creative and were not too much bounded by frameworks. Future research may explore how children can be more involved in the theoretical steps of IM.

## Supplementary information

**Additional file 1.** Steps of Intervention Mapping and how they are applied when combined with YPAR.

**Additional file 2.** Graphic implementation plan of the Olympic sports event.

**Additional file 3.** Graphic implementation plan of the cooking workshops.

## Data Availability

Data and materials accompanying this publication are available upon request.

## Abbreviations

Amsterdam UMC: Amsterdam University Medical Centers
IM: Intervention Mapping
YPAR: Youth-led Participatory Action Research
