# Supplementary material for: Co-designing obesity prevention interventions together with children: intervention mapping meets youth-led participatory action research
Source: Int J Behav Nutr Phys Act. 2019 Dec 12;16:130. doi: 10.1186/s12966-019-0891-5 (PMC6909512; doi:10.1186/s12966-019-0891-5)
Supplement: Supplementary file 1 — Additional file 1. Steps of Intervention Mapping and how they are applied when combined with YPAR. [file 12966_2019_891_MOESM1_ESM.pdf]

Additional file 1: Steps of Intervention Mapping and how they are applied when combined with YPAR

The IM process consists of 6 steps, each containing of several tasks (**bold**) and subtasks. When boxes are empty, the task is similar to the IM process without involvement of children.

	Description IM process	Application in IM+YPAR
**Step 1**	**Logic model of the problem.**	
Task 1	**Establish and maintain a planning group that includes program stakeholders and community members.**	The planning group includes program stakeholders and youth. Additionally, establish YPAR groups and an IM expert panel if needed.
	Create a project group: community, stakeholders.	When available, join an existing project group.
	Choose a logic model of the health problem with the work group. The planner decides which questions need to be answered in the needs assessment.	The YPAR groups decide on the research questions that direct the needs assessment.
Task 2	**Plan and conduct the needs assessment using the logic model.**	The YPAR groups conduct their own research to serve as input for the needs assessment.
	Conduct needs assessment with work group as subjects: qualitative and quantitative data and literature search. Specific data source is used for each question the research wants to answer.	The YPAR groups get capacity building workshops on how to conduct research. The needs assessment is conducted with the children from the YPAR groups as co-researchers. Together with the academic researchers, children choose which data source to use for each of their research questions. A literature search is conducted by the academic researchers to add to the YPAR groups' data.
	Incorporate results in the logic model together with the work group: Describe the priority population and its context, behavior, health problems and quality of life.	Researchers execute this step, cross-check by YPAR groups.
Task 3	**Describe the context for the intervention.**	
	Balance the needs assessment with an assessment of community capacity and environmental assets.	Incorporate in YPAR process and literature search.
	Identify a setting or the intervention.	
Task 4	**Link the needs assessment to program and evaluation planning by specifying desired program goals.**	Analyze data together with YPAR groups. Let the YPAR groups define the most important results. The desired program goals are defined by the researchers and cross-checked by the YPAR groups.
	Think about relevance and changeability of behaviors and environmental conditions.	Execute together with the YPAR groups.
**Step 2**	**Program outcomes and objectives – use the logic model of change.**	

Task 1	**State expected program outcomes for health behaviors and environmental conditions to improve health and quality of life.**	Execute together with the YPAR groups.
	Identify health-related behaviors based on needs-assessment.	
	State behavioral outcomes.	
	Identify environmental outcomes – interpersonal, organizational, community and societal.	
	State environmental outcomes.	
Task 2	**Subdivide behavioral and environmental outcomes into performance objectives.**	Execute with researchers and expert panel. Discuss POs with YPAR groups.
	Specify performance objectives for behavioral and environmental outcomes.	
	Validate performance objectives.	Validate with expert panel and discuss the children's POs with the YPAR groups.
Task 3	**Select important and changeable determinants of the health behavioral and environmental outcomes**.	Execute with researchers based on needs assessment and literature. Cross-check with expert panel and YPAR groups.
	Get a deeper understanding of the individuals and context to select determinants: ask questions, make a list of possible answers, validate these answers with literature and data.	
	Refine the list of determinants by rating them on relevance and changeability.	
Task 4	**Create a matrix of change objectives for each ecological level to be included in the intervention (individual, interpersonal, organizational, community, and social).**	Execute with researchers and expert panel.
	Select intervention levels and differentiate the intervention population.	Execute with researchers and expert panel.
	Construct matrices and write change objectives.	Execute with researchers and discuss with expert panel.
	Identify environmental agents and program implementers.	
Task 5	**Begin creating a logic model of change for the health promotion program.**	
	Use matrices of change objectives to develop instruments for program evaluation.	
**Step 3**	**Selecting theory-informed intervention methods and practical applications.**	Step 3 and 4 can happen simultaneous or iterative. Make sure that all tasks in step 3 before have been executed before making definite decisions in step 4.

Task 1	**Generate ideas for program themes, components, scope, and sequence with the planning group (participants and program implementers).**	Program ideas come from YPAR groups. Before this task, rank POs in YPAR groups.
	Generate program ideas. Think about themes, components, scope, sequence, how are you going to deliver the message.	YPAR groups generate program ideas and vote for ideas with most potential. The researchers discusses these ideas with organizations from project group.
	Draft a plan that describes the program.	Make in the YPAR groups.
Task 2	**Identify theory and evidence-based change methods to address program objectives.**	
	Reorganize the change objectives by determinants.	
	For environmental conditions, select possible change agents.	Ask YPAR groups who they identify as possible change agents.
	Review existing empirical evidence in the literature and review theories of change.	
	Choose theoretical methods for changing behavior.	Choose theoretical methods after completion of task 3.
Task 3	**Select or design practical applications.**	YPAR groups decide on practical applications for one or two ideas. Researchers make sure all program and change objectives are included.
	Relate program ideas to the priority population – appropriately use the theory.	Execute together with the YPAR groups and the YPAR groups ask feedback on the program ideas from their peers.
	Ensure that the final applications of methods (still) address (all) the change objectives.	Discuss with the YPAR groups if the final applications still match their ideas and the program goals.
	Start planning the process evaluation.	
**Step 4**	**Program production.**	Produce program components and materials. Let the YPAR groups conduct as much as possible; if necessary involve partners.
Task 1	**Refine the program structure and organization that were generated in step 3.**	
	Make sure your program can reach the intended population.	Discuss with YPAR groups how to make sure their peers are willing to participate in the intervention.
	Check budget and time constraints.	
Task 2	**Prepare production plans for the program materials so that they meet the program objectives and are aligned with the culture of the participants, parameters for use of their change methods and practical applications.**	Conduct together with YPAR groups and partners.

	Conduct formative research to make sure the production plans meet the participant’s characteristics and pretest prototypes of program materials.	
	Develop design documents to convey your message. This can be done by the planning group, but is often done together with externals such as creative consultants. Also review already existing materials.	Use input from the YPAR groups.
Task 3	**Draft messages and produce preliminary or prototype materials and protocols for the program.**	YPAR groups conduct as much as possible. If necessary involve partners but let children give input and feedback.
Task 4	**Pretest and pilot-test program components, and make revisions prior to overseeing materials’ production.**	Give children tasks in the execution of the pilots.
	Plan for program evaluation – contributions of pilot-testing and reception of program materials by participants.	
**Step 5**	**Program implementation plan – use dissemination and implementation frameworks.**	
Task 1	**Identify potential adopters and implementers of the health promotion program.**	Execute together with the YPAR groups.
	Reevaluate the planning group to ensure representation of potential program adopters and implementers.	
	Possibly create a linkage system between planning group and potential program planners and implementers.	The link is created. Communicate ideas or questions that come from the YPAR groups to program planners and implementers through the researchers.
Task 2	**State program outcomes and performance objectives for adoption, implementation, and maintenance.**	Discuss with the YPAR groups.
	Develop a plan to facilitate the adoption process.	Develop the plan in the YPAR groups, discuss with the program implementers and ask feedback from children outside the YPAR groups.
	Articulate what is required to implement the program and specify objectives required for program use.	
	Consider threats and facilitators of maintenance (institutionalization and sustainability).	YPAR groups and program planners/implementers think about threats they expect and incorporate these in the implementation plan.
Task 3	**Construct matrices of change objectives for program adoption, implementation and maintenance.**	Add input from YPAR groups throughout this task.
	Matrices combine performance objectives and determinants, which are factors that are likely to influence their performance.	

	The team will brainstorm over barriers and facilitators to accomplish performance objectives, in a comparable process as step 2: ask questions, make a list of possible answers, validate these answers with literature and new data.	
	Construct matrices for program use.	
Task 4	**Select change methods and practical applications, design the scope and sequence, and produce materials for an implementation intervention to influence program use.**	Add input from YPAR groups throughout this task.
	Comparable process to step 3, program planners use information from previous steps to select implementation intervention methods and practical applications.	
	Use selected change methods and practical applications to develop a deliverable intervention.	
**Step 6**	**Evaluation plan**	Start with this step as soon as the focus of the project becomes clear. It can take time before ethical clearance and consent is obtained.
Task 1	**Write process and effectiveness questions. These questions will come from a review of the program logic models, goals, and objectives and the IM matrices.**	Discuss potential youth-identified outcome measures with YPAR groups.
	Write effect evaluation questions based on the logic model of change: effect on health, quality of life, behavior, and environment; effect on change objectives; determine an evaluation time frame.	
	Write process evaluation questions for program implementation, appropriate operationalization of theories.	
Task 2	**Develop indicators and measures to assess the selected effect and process evaluation questions.**	If YPAR groups identified outcome measures, choose or develop suitable measurement tools.
	Develop measures suitable to the indicators.	
Task 3	**Specify designs for conducting process and effect evaluations.**	
	A suitable design for the process and effect evaluations is necessary.	Researchers make a planning, and include room to be flexible, especially in the process evaluation.
Task 4	**Specify and complete the evaluation plan.**	
	The plan includes the effect and process evaluation questions, primary outcome, indicators and measures, the design with the timing of the measures, and details about how the evaluation will be carried out.	
