# Supplementary material for: Co-designing obesity prevention interventions together with children: intervention mapping meets youth-led participatory action research
Source: Int J Behav Nutr Phys Act. 2019 Dec 12;16:130. doi: 10.1186/s12966-019-0891-5 (PMC6909512; doi:10.1186/s12966-019-0891-5)
Supplement: Supplementary file 2 — Additional file 2. Graphic implementation plan of the Olympic sports event. [file 12966_2019_891_MOESM2_ESM.pdf]

Organizational preconditions

Overall organization:
- Target group: children of grade 6/7/8 of four primary schools
- Organizing committee: 'Kids Aktief' and PAR groups
- Schools: PE teacher and person of contact (if not the PE teacher)
- Parents to assist at the final event
- Adolescents to assist at the final event

Input:
- Sports locations for both the trainings and final event
- Finances for the trainings and final event
- Room in calendar for trainings (can depend per school) and final event (same for all schools)

1

Implementation plan

Administrative level:
- Schools are offered to participate in the Olympics by 'Kids Aktief'

Institutional level:
Person of contact at the school:
- Coordinates when leaflets are distributed by PE teacher
- Instructs PE teacher what information needs to be given with the leaflet
- Instructs PE teacher to personally invite children who can benefit most from the extra sports activities
- Instructs PE teacher to make teams for the final event
- Informs teachers about the program
- Finds possible dates for the final event
- Informs teachers that they have to involve parents
- Gets sports materials and manuals for games that teachers can use during break time
- Coordinates with teachers where children can sign up for the trainings
- Makes a reservation for the sports location for the trainings
- Posts a message in the school's newsletter/website/email to parents

Executive level (trainings):
- Makes calendar for trainings
- Asks children what sports they want to do
- Makes a training schedule for 8 weeks
- Makes a schedule for the coaches; the same two coaches to one school
- Arranges sports material

Executive level (final event):
- Coordinates with the schools the date for the final event
- Makes reservations for the sports location
- Coordinates with the person of contact how many teams will participate
- Makes a schedule of all sports where schools compete against another school
- Arranges materials (sports, fruit, water) and personnel (sports, first aid, host, adolescents)
- Coordinates the day schedule and rules with person of contact

2

Program

Training
- All children of grades 6/7/8 may participate, children who can benefit most from extra sports activities are personally invited by their PE teacher
- The main goal is that all children have a positive sports experience
- In the trainings children can practice the sports of the final event
- At the beginning of each training the sports and rules are explained. These are also the rules at the final event.
- In one training the school flag is made.
- Children are corrected on their behavior and can get a yellow or red card for negative behavior. These are also the rules at the final event.
- At the end of the training the rules of each sport will be repeated.
- If children like a sport, they will get information about the sports club.
- Information on alternative finances for sports participation are given to children and their parents

Final event
- All children of grade 6/7/8 have to participate
- Schools compete against each other
- At the start all children are thanked who helped to host the event and each school is welcomed by their flag
- The event starts with an opening dance
- The scores (of sports and fair play) are monitored on a large screen
- During the day the coaches motivate children to play for the Fair Play cup
- Every child wins a prize
- Two cups are rewarded: one for fair play, one for achievement

Abbreviations:
PAR: Participatory Action Research
PE: Physical Education
MVPA: Moderate to Vigorous Physical Activity

3

Strategy
For children
- Practice of all sports
- All children have a positive sports experience because of the wide range of sports and activities on offer
- Group building:
  - School versus school
  - Opening dance and making a flag
  - Two cups can be won with the school: one for fair play, one for achievement
- Reward positive behavior
- 'Punish' negative behavior
- The coaches make sure all children get a feeling of success
- The coaches enthuse children for the sports activities
- Adolescents are used as referees/group leader
- Children co-decide on the program plan

Strategy
For schools
-Sports material and manual for games

Strategy
For the project group
- Promotion material
- Good relations with the schools and children
- Create a positive sports environment for children

4

Methods
- Forming coalitions
- Mobilizing social networks
- Punishment
- Reinforcement
- Team building and human relations training

- Discussion
- Rehearsal

- Forming coalitions
- Mobilizing social networks
- Punishment
- Reinforcement
- Team building and human relations training
- Use of lay health workers; peer education

- Guided practice
- Mobilizing social networks
- Verbal persuasion

5

Determinants of the program goal: more children participate in sports activities (children's level)

Skills + self-efficacy
- Children participate in a sports activity independent of where their friends are going
- Children demonstrate pointing other children to negative behavior
- Children demonstrate confidence in participating in sports activities independent of their friends

Knowledge
- Children know the sports activities that they prefer
- Children know the sports activities that are on offer
- Children know alternative financial possibilities to cover their sports participation

Perceived social norms
- Children recognize that peers participate in sports activities of their own preference

Attitude
- Children like participating in sports activities

6

Behavior
Higher levels of physical activity:
- Increase in total moderate-to-vigorous physical activity
- Increase in moderate-to-vigorous physical activity in bouts
- Decrease in sedentary time
- Decrease in sedentary time in bouts
- Increase in time spent playing outdoors

7

Intended result
At least 65% of 9-12 year old children of the participating schools adhere to the Dutch physical activity guidelines (i.e. 60 min MVPA per day)
