# Supplementary material for: Co-designing obesity prevention interventions together with children: intervention mapping meets youth-led participatory action research
Source: Int J Behav Nutr Phys Act. 2019 Dec 12;16:130. doi: 10.1186/s12966-019-0891-5 (PMC6909512; doi:10.1186/s12966-019-0891-5)
Supplement: Supplementary file 3 — Additional file 3. Graphic implementation plan of the cooking workshops. [file 12966_2019_891_MOESM3_ESM.pdf]

Organizational preconditions

Overall organization:

Target group: children of grade 6/7/8

School:
- Teachers of grade 6/7/8
- Person of contact

Community partners:
- Chef
- Local government
- Community organizations
- Local grocery store (sponsor)

Input:
- Chef
- Finances and/or sponsors
- Kitchen, materials and products

1

Implementation plan

Administrative level:
- School is offered the workshops through the local government and community organizations

Institutional level:
Personal of contact at school:
- Coordinates with the teachers that the promotional leaflets are handed out, what has to be explained and where the leaflets have to be handed in
- Explains the program to teachers, parents and children
- Writes an item in the school newsletter, website and emails parents

Executive level (chef):
- Prepares the program including the recipes
- Makes sure the recipes and products match the cultures of the children
- Makes reservations for the kitchen and materials
- Aligns with local grocery store which products are needed

Executive level (project group):
- Designs leaflets and recipe book that will be handed out to all children of grades 6/7/8
- Brings leaflets to the schools
- Makes the groups for the workshops
- Calls parents to let them know their child is or is not in a group
- Asks parents to assist at the workshops
- Coordinates with the chef the handbook, recipes and teaching methods
- In charge of finances and/or sponsors

2

Lesson 1
- Children receive their own recipe book
- Children learn three types of breakfast
- Children are encouraged to make each type of breakfast at home this week

Lesson 2
- Children learn three types of lunch
- Children are encouraged to make each type of lunch at home this week

Lesson 3
- Children go to a community garden to get a tour from the manager
- When they are back in the kitchen they discuss what they have learned
- With the vegetables from the garden the children learn to prepare 3 types of evening meals
- Children are encouraged to make each type of evening meal at home this week

Lesson 4
- Children learn three types of healthy snacks
- Large numbers of snacks are made so children can share them in class the next day
- Children are encouraged to make the snacks at home, share them with peers and parents and share the recipe

3

Program
Overall:
- Chef gives instructions on healthy nutrition
- Children learn about new products
- Children receive their own recipe book
- Children learn 3 healthy meals per class
- Children are actively involved in preparing the meals
- The chef gives instructions on how to prepare the meals
- Children learn what appropriate portion sizes are
- Children explain to their parents what they have learned
- Children are encouraged to practice their skills and meals at home
- Photos and videos are made during the classes
- Children learn how they can make water and tea taste better

4

Strategy
For children
- Information about products, portion size and recipes from the chef
- Information about where products come from by the manager of the community
- Instructions on and show how to prepare meals
- Practice with preparing meals
- Working in small groups with multiple assistants
- Participants are encouraged to share their experiences with peers and parents
- Videos are made during the classes and shared with the school
- Schools are asked to show the videos in class
- At the end of the class a recap of everything that has been learned
- Children are encouraged to prepare the meals at home
- At the end of the class there is a joint meal with children and parents

Strategy
For the project chef
- Handbook
- Recipes
- Teaching methods

Strategy
For the project group
- Make and spread the videos
- Communication plan

5

Methods

- Active learning
- Guided practice
- Individualization
- Modeling
- Mobilizing social support/networks

- Active learning
- Consciousness raising
- Discussion
- Modeling

- Consciousness raising
- Cooperative learning
- Cultural similarity
- Mobilizing social support/networks
- Resistance to social pressure
- Tailoring

- Modeling
- Belief selection

6

Determinants of the program goal: less children eating energy-dense snacks (children's level)

Skills + self-efficacy
- Children demonstrate that they can make their own breakfast
- Children demonstrate the confidence that they do not have to eat unhealthy snacks to be cool
- Children demonstrate when they have eaten enough
- Children demonstrate that they are capable of choosing for healthy snacks

Knowledge
- Children know different kinds of healthy breakfast
- Children know the importance of a healthy breakfast
- Children know what healthy snacks are
- Children know different kinds of products and which are healthy
- Children know what a normal portion size is
- Children know that it is not healthy to eat unhealthy snacks every day

Perceived social norms
- Children recognize that peers have a healthy breakfast
- Children recognize that other children eat healthy snacks
- Children recognize that children are not being made fun if when they eat healthy snacks
- Children recognize that fruits and vegetables are a snack

Attitude
- Children have positive feelings towards having a healthy breakfast every morning
- Children have positive feelings towards healthy snacks
- Children have positive feelings towards sharing a large unhealthy snack

7

Behavior
Less consumption of energy-dense snacks due to:
- More children having an appropriate amount of healthy breakfast
- Children eat fruits and vegetables as snacks

8

Intended result
At least 50% of 9-12 year old children of the participating schools consume a maximum of 1 portion (i.e. 1 bar) of snacks per day that are rich in sugar, salt, or fat
